# CellTrackVis: interactive browser-based visualization for analyzing cell trajectories and lineages

**DOI:** 10.1186/s12859-023-05218-y

**Published:** 2023-03-29

**Authors:** Changbeom Shim, Wooil Kim, Tran Thien Dat Nguyen, Du Yong Kim, Yu Suk Choi, Yon Dohn Chung

**Affiliations:** 1 School of Electrical Engineering, Computing and Mathematical Sciences, Curtin University, Perth, Australia; 2 Data Intelligence Team, Samsung Research, Seoul, South Korea; 3 School of Engineering, RMIT University, Melbourne, Australia; 4 School of Human Sciences, University of Western Australia, Perth, Australia; 5 Department of Computer Science and Engineering, Korea University, Seoul, South Korea

**Keywords:** Cell tracking, Data visualization, Cell trajectory, Cell lineage

## Abstract

**Background:**

Automatic cell tracking methods enable practitioners to analyze cell behaviors efficiently. Notwithstanding the continuous development of relevant software, user-friendly visualization tools have room for further improvements. Typical visualization mostly comes with main cell tracking tools as a simple plug-in, or relies on specific software/platforms. Although some tools are standalone, limited visual interactivity is provided, or otherwise cell tracking outputs are partially visualized.

**Results:**

This paper proposes a self-reliant visualization system, CellTrackVis, to support quick and easy analysis of cell behaviors. Interconnected views help users discover meaningful patterns of cell motions and divisions in common web browsers. Specifically, cell trajectory, lineage, and quantified information are respectively visualized in a coordinated interface. In particular, immediate interactions among modules enable the study of cell tracking outputs to be more effective, and also each component is highly customizable for various biological tasks.

**Conclusions:**

CellTrackVis is a standalone browser-based visualization tool. Source codes and data sets are freely available at http://github.com/scbeom/celltrackvis with the tutorial at http://scbeom.github.io/ctv_tutorial.

**Supplementary Information:**

The online version contains supplementary material available at 10.1186/s12859-023-05218-y.

## Background

Visually analyzing cellular behavior is crucial in obtaining remarkable insights into developmental biology. Specifically, understanding cell movements is one of the paramount biological tasks, and interpreting cell lineages is also nearly equal. In this regard, visual analysis of cell behavior is considered as two phases: *Tracking* and *Visualization*. Computational cell tracking is generally a substantive solution to determine cell trajectories with genealogy [1, 2], whereupon various cell tracking methods have been steadily developed [3–8]. However, visualization for tracking outputs still has room for further improvements. Common visualization tools, usually components of the main cell tracking software, are not fully independent [6, 7, 9, 10]. Although some advanced works [11–14] introduced improved visualization, they are platform/software-dependent (as plugins or toolboxes) with certain prerequisites, installation, or licenses, and may not be suitable for a wide range of users. There are a few tools dedicated solely to visualizing the output of cell tracking methods regardless of specific preparatory platforms or software. In [15], an interactive 2D visualization of cell lineages was devised without displaying cell images. This browser-based tool is self-reliant but is no longer available. CeLaVi [16] is a state-of-the-art web-based system for interactive cell lineage visualization. The analysis tool of cell trajectories is excluded even though its interworking panels show 3D cell coordinates with ancestries. Arguably, it is necessary to develop an easy-to-use method that is able to support interactive visualization of cell tracking outputs with the coverage from standalone open-source software to online services.

In this paper, we propose CellTrackVis, an independent visual analysis system, which allows users to conveniently analyze live cell activities. Without any further installation or download, users simultaneously perform their interactive visualization through standard browsers by accessing a designated website. This convenience provides a quick start, especially for non-tech-savvy practitioners, and it also allows the users to perform analysis on common platforms with no requirement of preliminary software. Meanwhile, a host (server) can provide web services by freely customizing our code. If someone wants to run and use it privately, it is also possible on local machines. Specifically, CellTrackVis visualizes tracking results, e.g., cell trajectories, segmentation, raw or processed image sequence, cell lineages, or quantified information, on interconnected views. In addition, our open-source software is flexibly developed, wherein source codes can be customized easily.

## Implementation

### Visualization system requirements

We establish desirable goals for addressing visual analytic tasks in cell tracking results. Biologists generally require a seamless analysis process in successive research steps. Thus, the below implementation guide is based on the opinion of domain experts.

**R1**: Visual interactivity
  Interaction features are effective in exploring tracking results throughout tool interfaces. When visualized trajectories are selected by users, it enables clonal lineage tracing and subsequent sub-clonal analysis, e.g., comparison of migration trend and proliferative capacity based on clonal origins. Mouse-hovering on cell points or lineage trees with following immediate reactions is also invariably helpful.
**R2**: Standalone tool
  Despite the rapid development of software for bioinformatics, learning to use existing platforms with their functions is one of the major obstacles. This issue worsens when they just need to simply utilize new or existing tools for making quick decisions. A long analysis pipeline consists of various experiments, and eventually, many users finish tasks manually for moving on by giving up becoming proficient at the platforms. In other words, simple standalone software is arguably crucial to quick and easy analysis.
**R3**: Integrated interfaces
  Coordinated visualization views are imperative and should be implemented to be properly fitted to a screen. Independently designed modules sometimes distract users due to overlapping pop-up windows or uninstalled packages yet. By providing synchronized panels, unified interfaces help users discover eye-catching cell movement and behavior changes instantly.
**R4**: Flexible input
  Various cell trackers usually generate trajectories and/or lineages with similar objectives. Other pertinent information like the numbers of cell appearance, disappearance, or division can be also acquired by some recent algorithms. Furthermore, plotting raw or processed images is reasonably practical in its analysis. Last but not least, cell tracking outputs are needed to be visualized regardless of data composition and independently among modules in the system for smooth and expeditious visual analytics.

Note that detailed descriptions in the next subsection are followed by relevant requirement marks from **R1** to **R4**.

**Fig. 1:**
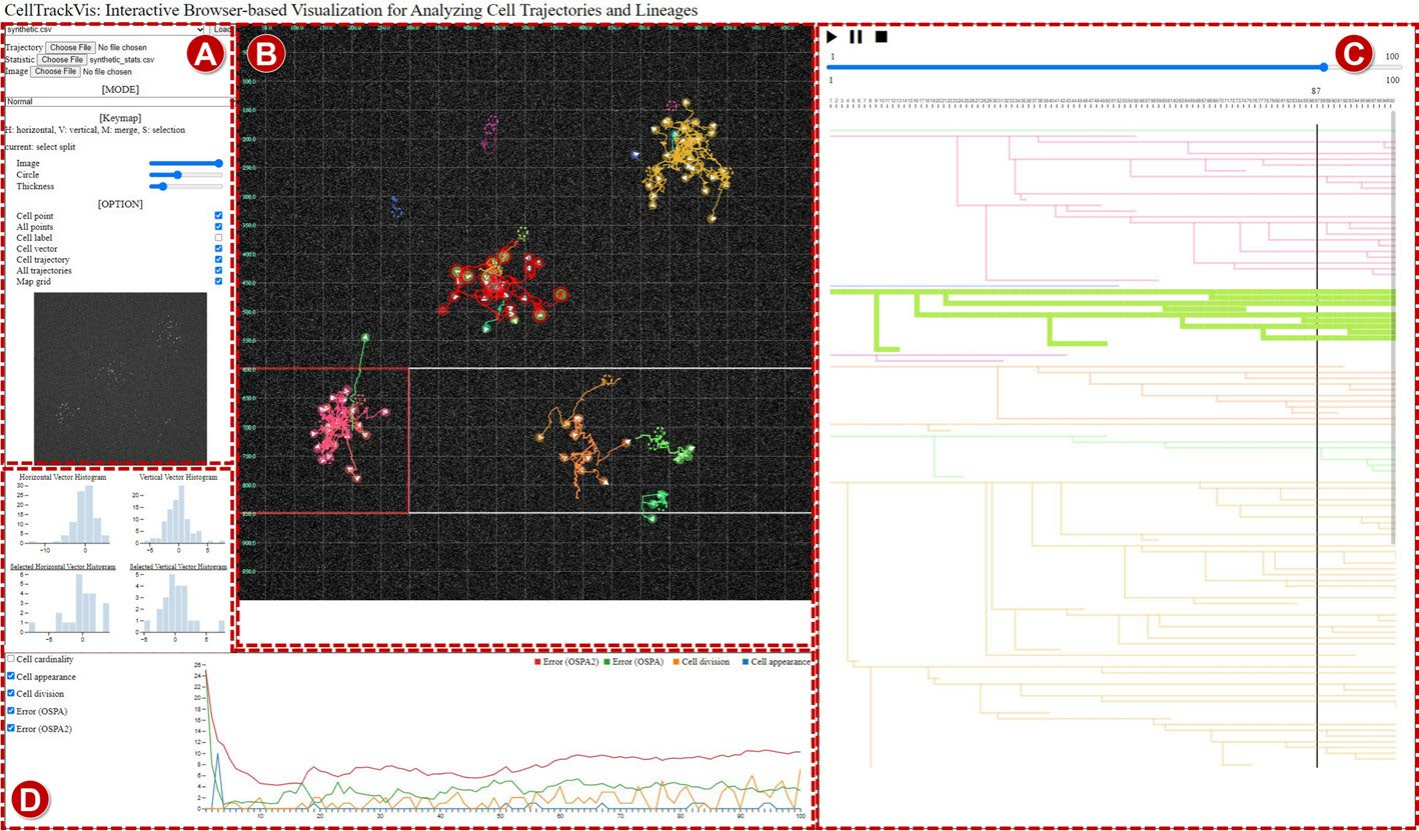
The overview of CellTrackVis: **A** Setting view; **B** Trajectory view; **C** Lineage view; and **D** Statistic view

### System design

**Fig. 2:**
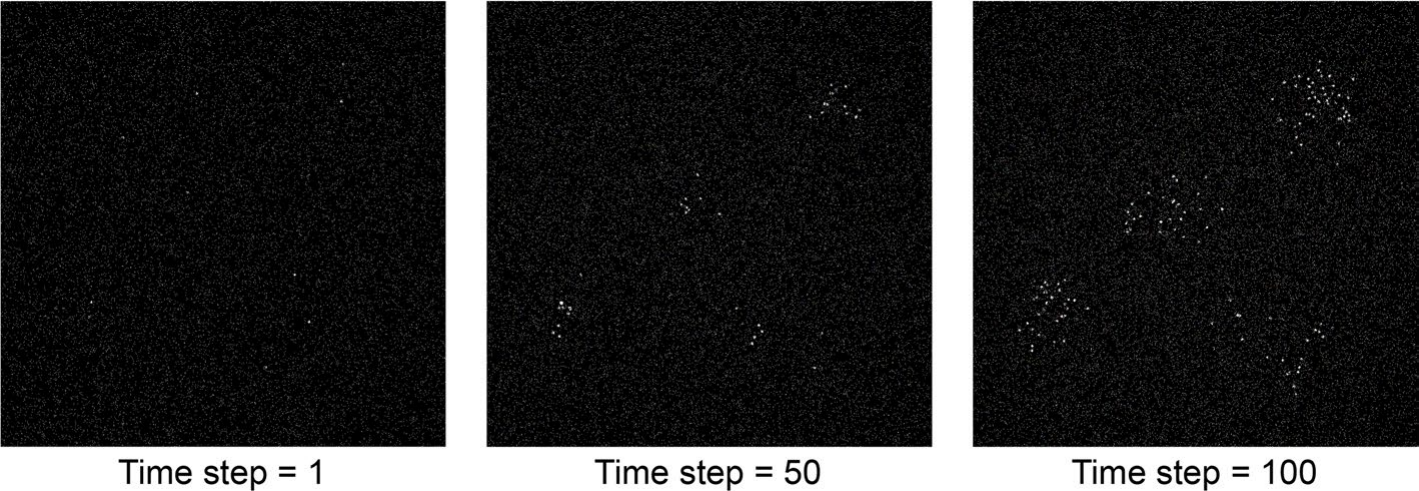
Snapshots of synthetic cell image sequence [17] of 1000 x 1000 pixels at different time instances

Using CellTrackVis, users visually analyze their cell tracking results via common web browsers without any critical dependency of platform and software (**R2**). The interactive visualization system is composed of four interlocking modules (**R1**, **R3**): **A** *Setting view*; **B** *Trajectory view*; **C** *Lineage view*; and **D** *Statistic view*. Figure 1 shows the overview of the proposed tool on the synthetic data [17] of which snapshots are depicted in Fig. 2. Our tool is optimized for display with 5120 $\times$ 2880 resolution, and the best-fit size of the interface can be found by zooming in or out without affecting visualization quality.

CellTrackVis is implemented with Python and JavaScript libraries like D^3^ [18], and source codes are freely available on the GitHub page. Once a Python server runs, celltrackvis.html handles all functions for visual analysis. Specifically, main_pro processes and visualizes the default synthetic data set (Fig. 2) until new data are provided, and Update updates every entire screen. Additionally, UpdateHistogram manages two histograms in the setting view, and both UpdateSplitHistogram and current_detail_mode are related to Split mode (explained in the subsection of space partitioning). Although the provided simple web server file is adequate for research purposes or private use, some popular frameworks such as Flask [19] and others can be employed for providing professional services. For more details, readers are referred to the tutorial web page.

**A** Setting view

**Fig. 3:**
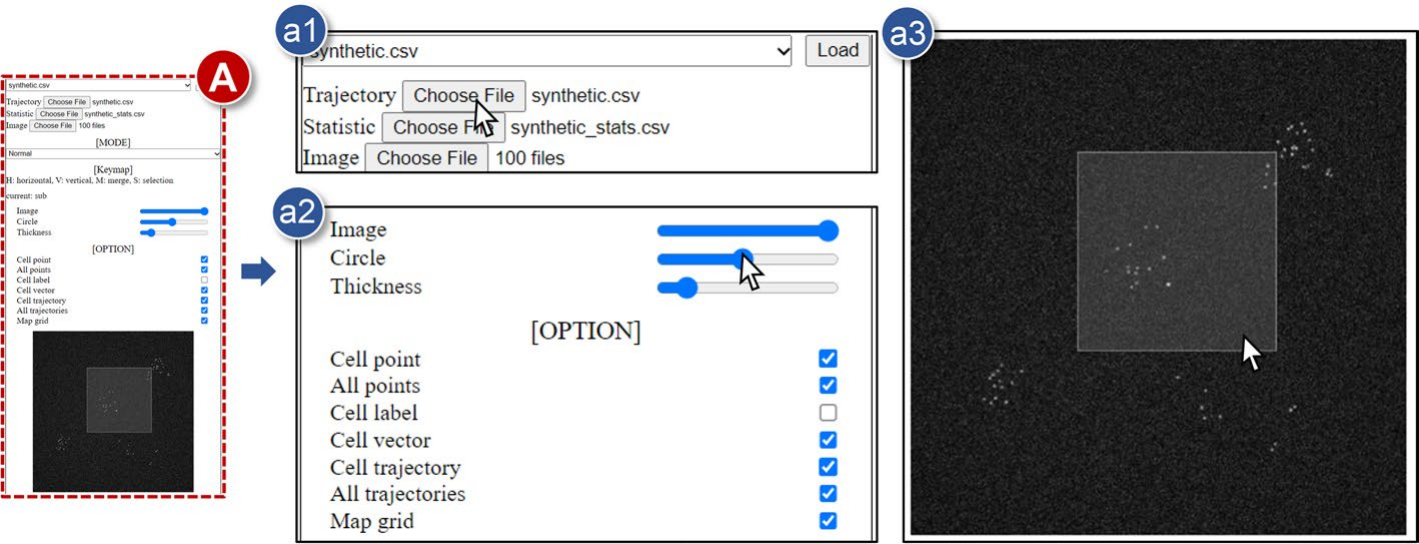
Details of the setting view (**A**): **a1** Data upload; **a2** Option controller; and **a3** Mini map

Practitioners can visualize their data generated from cell trackers and set several options linked to the trajectory view via this module (Fig. 3).

**a1**: *Data upload*
  CellTrackVis accepts tracking outputs as inputs such as trajectory, lineage, quantified information and background image, respectively (**R4**). We adopt the well-known file format of Comma-Separated Values (CSV) for a wide range of users. First, trajectory with lineage file format is composed as follows [time step, ID, left, top, height, width, img_height, img_width, parent], where time step denotes the time step of trajectories, ID is a unique positive number of the cell, left/top/height/width is respectively the minimum x coordinate, the maximum y coordinate, the height/width of the detection for the cell, img_height/img_width is the height/width of the entire (background) image, and parent denotes the parent ID of the cell. The input form of the bounding box is inspired by the general Multiple Object Tracking (MOT) challenge^1^ by further considering the characteristic of cell ancestry. This approach is not only simple and intuitive but also flexible. In other words, common techniques in various fields are compatible with CellTrackVis. In computer vision, for example, general object detection or tracking methods are based on the bounding box, and the rectangular approximation effectively tackles computational bottlenecks in databases and data mining. Our tool is designed for users with different levels of experiences and backgrounds, and the straightforward box clearly benefits non-tech-savvy users who want quick and easy analysis. Moreover, service providers using the proposed tool can simply change the shape by considering their purposes. Since our software adopts box form inputs, if users’ cell trackers can generate only other formats, e.g., 2D coordinates, they should be converted to boxes with some reasonable height and width using our importer. A dummy number $(-1)$ can be used for filling in blanks when users want to ignore lineage data or cell trackers cannot generate it. The proposed tool also supports Cell Tracking Challenge (CTC) data format, which is widely used by state-of-the-art cell trackers, through default importers. In subsequent releases, we will include additional importers for new types of formats when their popularity emerges in the literature. While tracking cells, some algorithms quantify useful information such as cell mitosis, appearance, and disappearance in each time step. These time-series data can be a kind of CellTrackVis inputs if needed in the analysis. The statistic file consists of as follows [time step, 1st stat., 2nd stat., 3rd stat.,...], where time step denotes the time step of lineage data, and 1st/2nd/3rd/... stat. denotes the first/second/third/... quantified information respectively. Table 1 describes an example of the statistic file. Finally, plotting a sequence of 2D/3D images is also available. Users decide whether image backgrounds are loaded in advance or on the fly according to their objectives, because image inputs are not necessarily needed in CellTrackVis. Although visualization views are interrelated, each component of our tool can be independently utilized, e.g., trajectory data only, or trajectory with image backgrounds. CellTrackVis accepts generally known image file formats: JPEG; PNG; BMP; GIF; and TIFF. In particular, a 2D image is plotted at each time step, and a 3D (multi-channel) file is handled in a slice view manner (as a 2D image sequence for each time point). Other formats also will be supported in subsequent releases for users’ convenience. The corresponding images should be provided in the same order of input trajectories or lineages for proper use without any other data processing. Here, image processing time and memory usage depend on server machines and are controlled by a host.
**a2**: *Option controller*
  The general setup of the trajectory view is available in this panel (**R1**). Specifically, people can intuitively control data visibility via the following three control bars: the bar Image for the transparency level of background images; the bar Circle for the size of cell points; and the bar Thickness for the thickness of cell trajectory lines with vectors of direction. In particular, the controller of image transparency is useful when users want to concentrate on analyzing data by adjusting the background transparency on the fly. Note that the background (image) is not mandatory because cell trajectories and lineages can be visualized and analyzed without corresponding images in the proposed method. Notwithstanding this independence, plotting raw image sequences (or estimated segmentation masks) is immediately helpful in studying cell shapes with trajectories/lineages simultaneously. Furthermore, tracking outputs can be selectively plotted by seven checkboxes: the toggle Cell point for cell positions, which have not disappeared; the toggle All points for all cell positions; the toggle Cell label for cell labels composed of an identifier and the birth time; the toggle Cell vector for vectors of cell direction to the next location; the toggle Cell trajectory for linked lines between previous and current positions of cells, which have not disappeared; the toggle All trajectories for dotted linked lines between previous and current positions of all cells; and the toggle Map grid for grid lines on the entire area. Last but not least, splitting the monitoring space is possible by changing the mode from Normal to Split. Details of this attractive function are described in sections of the trajectory view, the statistic view, and a usage scenario.
**a3**: *Mini map*
  A downsized map is presented with this module as an overview of the trajectory view. Users are able to simultaneously explore both local and global spaces using this component. First, its range is zoomed in and out by scrolling a mouse up and down, respectively (**R1**). Then, the region of interest in the current trajectory view matches with a small gray rectangle of this mini map. The monitoring area moves through mouse clicks and drags.

**Table 1:** The statistic data format

Time step	No. cells	No. mitosis	No. appearance	MSE	$\cdots$
1	3	0	3	0	$\cdots$
2	5	2	0	4	$\cdots$
3	7	0	2	1	$\cdots$
$\vdots$	$\vdots$	$\vdots$	$\vdots$	$\vdots$	$\ddots$

**B** Trajectory view

**Fig. 4:**
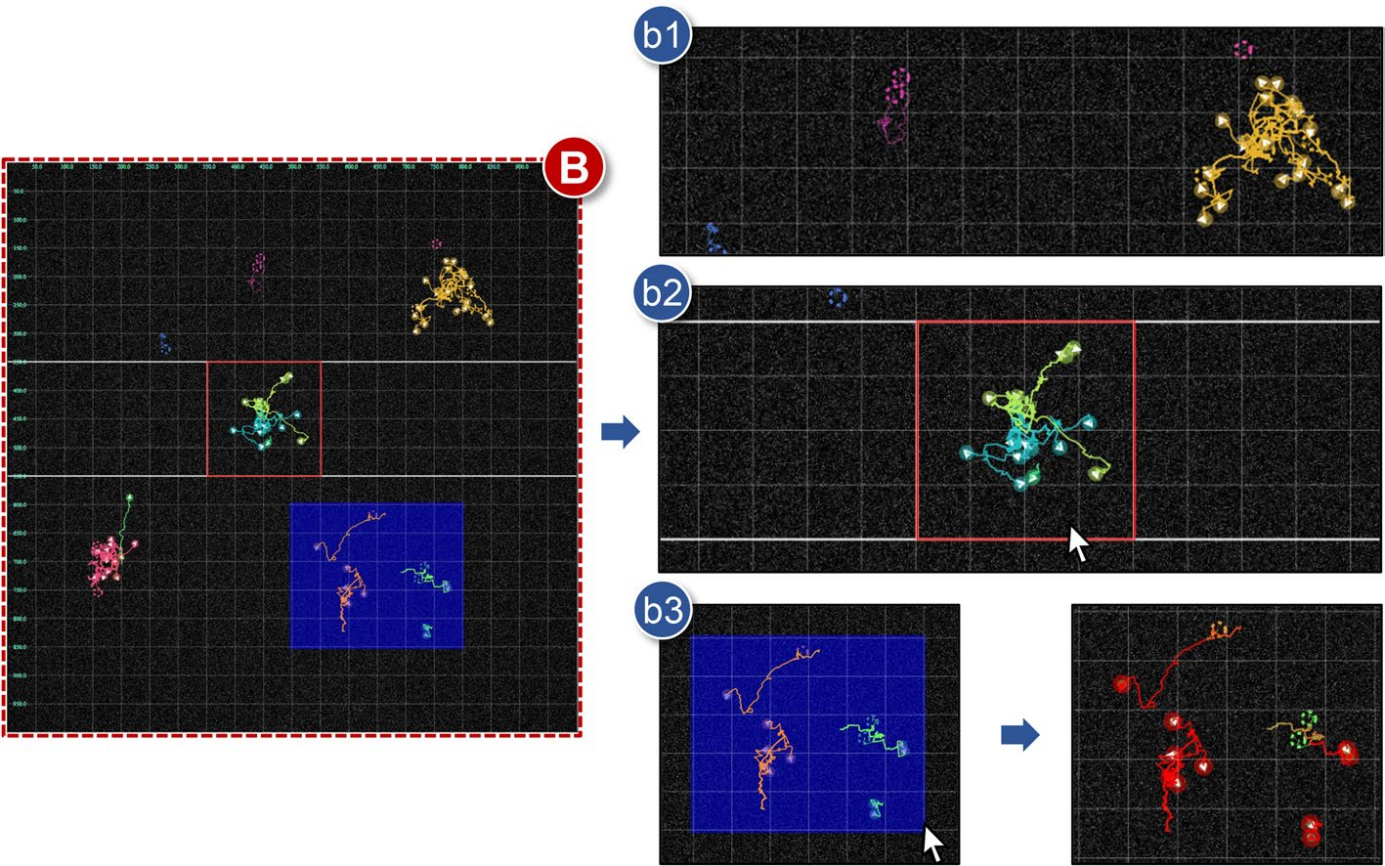
Details of the trajectory view (**B**): **b1** Visibility control; **b2** Space partitioning; and **b3** Trajectory selection

Displaying cell trajectories is the main objective of this panel (Fig. 4). Data of interest could be analyzed in depth with respect to cell point/trajectory and (raw) images. Furthermore, various visualizations are practicable via the combination of settings.

**b1**: *Visibility control*
  Circles and arrows express present cell positions and vectors of direction to the next position. The default setting is the location (solid circles) and trajectory (solid lines) visualization of cells currently living. Through checkboxes All points or All trajectories in the setting view, those of disappeared cells also can be plotted by dotted circles or lines. Other options for cell labels, vectors, and a map grid are intuitive yet helpful. The size of circles, the thickness of lines, and the level of background transparency are adjusted using bars of the setting view. Moreover, users can zoom in or out the areas where they are interested in by scrolling a mouse up or down. Our tool can seamlessly handle a large number of cells owing to the efficient SVG, JavaScript libraries and the developed zooming functionality. Further, since the raw tracking image can be displayed in the background with an optional transparency level, users can visually examine the morphology of the corresponding cells. Future development will also incorporate cell morphology analysis to facilitate more analysis workflows, given that morphological data are also available (e.g., imported as CTC format).
**b2**: *Space partitioning*
  In addition to visibility change, the entire field is further divided into several zones for detailed analysis. Analysts can partition the whole region off after choosing Split mode. Each function in the mode is chosen by using the following keyboard shortcuts: the key H for adding a horizontal line; the key V for adding a vertical line; the key M for merging two sections by removing lines; and the key S for selecting a divided zone. When users select an interesting region, histograms of horizontal and vertical vectors in the boundary are additionally presented in the statistic view. In particular, subdivided regions are kept during other analyses if the mode returns to Normal. This space partitioning function is especially useful when there are lots of cells or hot spots in the monitoring area continuously.
**b3**: *Trajectory selection*
  Selecting trajectories is freely available for a single cell or multiple cells. When hovering a mouse cursor over any current cell point, it is emphasized, and then when clicking it, all points and trajectories of relevant family cells are colored as red (**R1**). A rectangular box is utilized to choose different cell families at once by clicking and dragging mouse in the trajectory view. Furthermore, lineages of the selected cells are simultaneously highlighted in the lineage view, and those vector histograms also appear in the statistic view.

**C** Lineage view

**Fig. 5:**
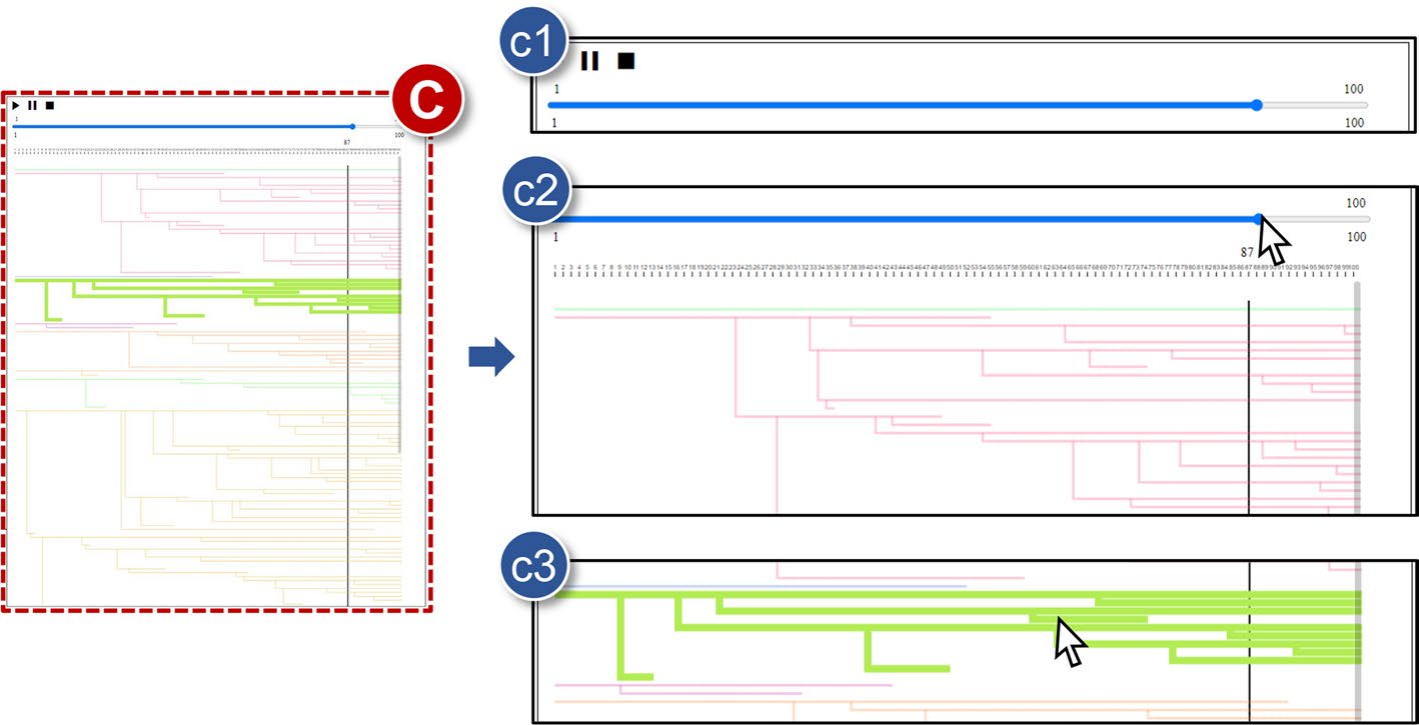
Details of the lineage view (**C**): **c1** Animation player; **c2** Time bar; and **c3** Lineage selection

Time-varying cell ancestries are presented on this interface (Fig. 5). We can especially analyze cell behaviors in general or step by step using either convenient animation functions or a control bar, respectively.

**c1**: *Animation player*
  Visualized tracking outputs can be played like a video. When users click a play icon (►), most graphics in trajectory, lineage, and statistic views are played back simultaneously (**R1**, **R3**). For example, cell trajectories, direction vectors, and background images are animated in the trajectory view. Furthermore, histograms in the setting view and a vertical line bar in the lineage view continuously change at each time step with the animation in the trajectory view. Basic pause icon  and stop icon (■) are also essential for this function.
**c2**: *Time bar*
  A time bar helps us to delve into the time-varying movements of cells one by one. Time steps are controlled by the hand-operated fashion using the bar, and cell divisions can be easily identified thanks to a moving vertical line across all lineages. Moreover, this feature is directly related to cell trajectories because the state of the trajectory view changes interactively when moving the time bar forward (left) and backward (right) (**R1**). Histograms in the statistics view also have similar visualizing mechanisms. In other words, time-varying changes in cell tracking results are explored in the proposed tool using the animation operation or this manual bar, depending on the scenarios.
**c3**: *Lineage selection*
  It is important to study spatio-temporal characteristics of live cells with their pedigree. A lineage tree can be hovered and chosen by using a mouse pointer on account of its usefulness. In particular, the trajectory view stresses the corresponding cell points concurrently by sizing them up whenever hovering over any lineage structure. When the tree is selected by clicking it, the emphasis is further placed via coloring not only the outline of cell points but also trajectories at once as red. Therefore, users are able to figure out which cell family has the hovered or selected ancestries (**R1**).

**D** Statistic view

**Fig. 6:**
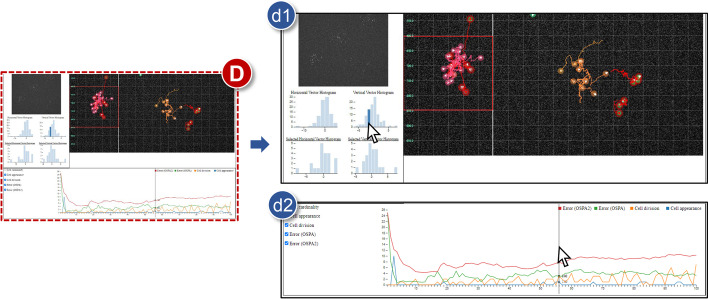
Details of the statistic view (**D**): **d1** Vector histogram; and **d2** Time-series statistic

This panel shows various quantified information during cell tracking (Fig. 6). Particularly, histograms for (vertical or horizontal) direction vectors of moving cells are presented here for both whole and selected areas. Line graphs are also plotted for dealing with general statistics in tracking output data.

**d1**: *Vector histogram*
  The statistical analysis of direction information in cell motions is significant. For its visual exploration, a histogram is provided regarding horizontal degrees versus global populations of current cells. Analogously, vectors of vertical directions are also considered in the same way on another histogram. Then, analysts can carefully check the number of cells moving horizontally or vertically with their heading angles at each time step. Furthermore, straightforward interactions enable us to find relevant trajectories and ancestries by matching other views. They are accentuated whenever a related bar in the histogram is selected. In addition to the entire region, the above functions can be identically performed in the selected section. Two histograms appear below the global histograms after splitting the trajectory view using Split function and selecting a region of interest using the shortcut of the selection.
**d2**: *Time-series graph*:
  CellTrackVis visualizes not only trajectories or lineages but also other quantified information generated by some cell trackers. Those generally include the number of cell division or appearance/disappearance at each time step. Distinct time-series data are plotted using line graphs and exact values appear with a vertical bar, moved by a mouse pointer. The statistic data set is not the mandatory input, and thus our tool supports its visual analysis while retaining the flexibility of input data (**R4**). It is also available to turn on and off line graphs via checkboxes similar to the part for the trajectory view. Sometimes line graphs can be overlapped, and it results in a complicated analysis process if the statistic file has large cardinality, i.e., the number of columns. In the statistic view, however, users can load all data at once and then check the graphs one by one using checkboxes.

## Results and discussion

### Usage scenario

**Fig. 7:**
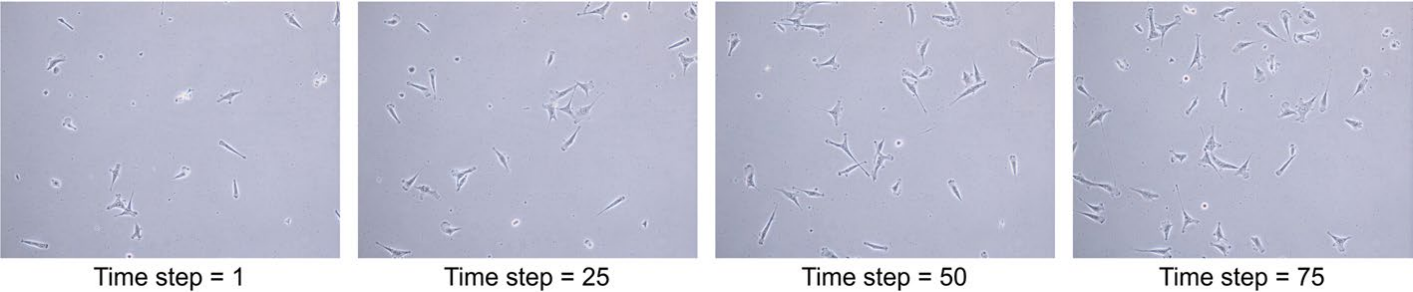
Snapshots of breast cancer cells (MDA-MB-231)

**Fig. 8:**
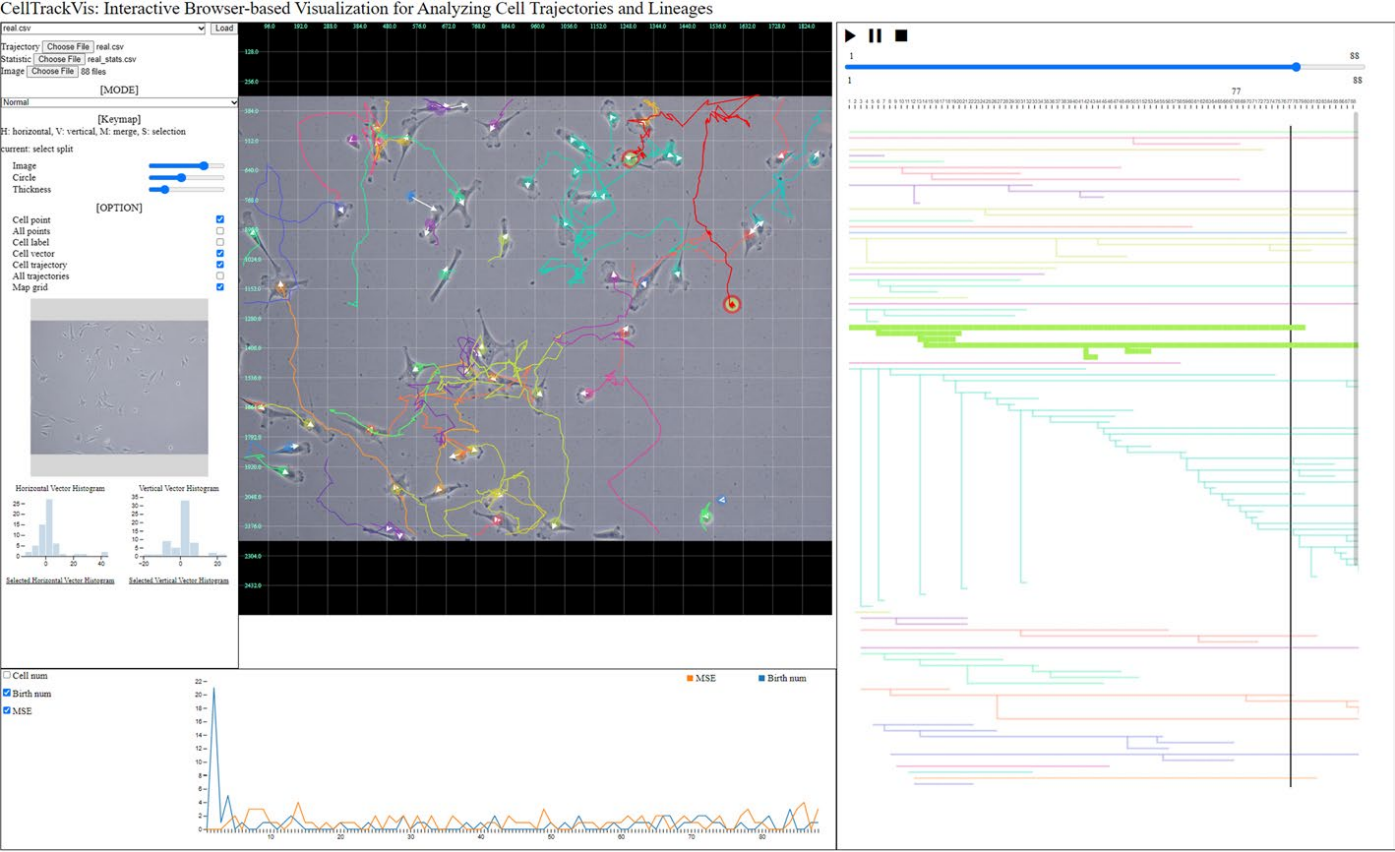
Usage scenario on real data set

This section demonstrates our system for analyzing the behaviors of breast cancer cells, MDA-MB-231 (Fig. 7). We captured the time-lapse of cell migration with the resolution of 2560 $\times$ 1920 pixels at the rate of one frame per 15 min. We first supplied the sequence of images to a state-of-the-art cell tracker [8] for obtaining the tracking results (motion, lineages, and other statistics). Those visualizations were then performed using CellTrackVis. We tested our tool as a local machine user in Chrome browser (108.0.5359.126 (64-bit)) on Intel(R) Core(TM) i5-8250U CPU @ 1.60GHz with 16GB memory. A screenshot during the analysis is plotted in Fig. 8. Note that additional visualizations for other data sets (cell tracking challenge^2^) are also included in a Additional file 1, where 1,700 images (BF-C2DL-HSC) and 650 cells (PhC-C2DL-PSC) are highlighted.

From the setting view, we imported the tracking results, CSV files containing cell trajectories, lineages, other data, and images. After adjusting point size, line thickness and image transparency, cell trajectories were displayed in the trajectory view. Note that because we display the raw tracking image in the background, the transparency option allows us to adjust to either focus on the tracking results (motion patterns) or the actual cells on the image (morphology). Further, since each trajectory was highlighted with a distinct color, we can visually trace its motion pattern in a convenient way. This analysis can be performed seamlessly even when cell density is high since users can zoom in or out a particular region. Moreover, the lineage view presented all ancestry trees at once. We conveniently navigated the lineage of a particular cell family by clicking on its trajectory, which was automatically and instantly highlighted the corresponding lineage tree in the lineage view. The statistic view showed us the statistics of the cell population at each time step in terms of the numbers of mitosis (spawns) and cells entering the region (births), and the overall velocity of cells, where cell tracking results contained the quantified information.

**Fig. 9:**
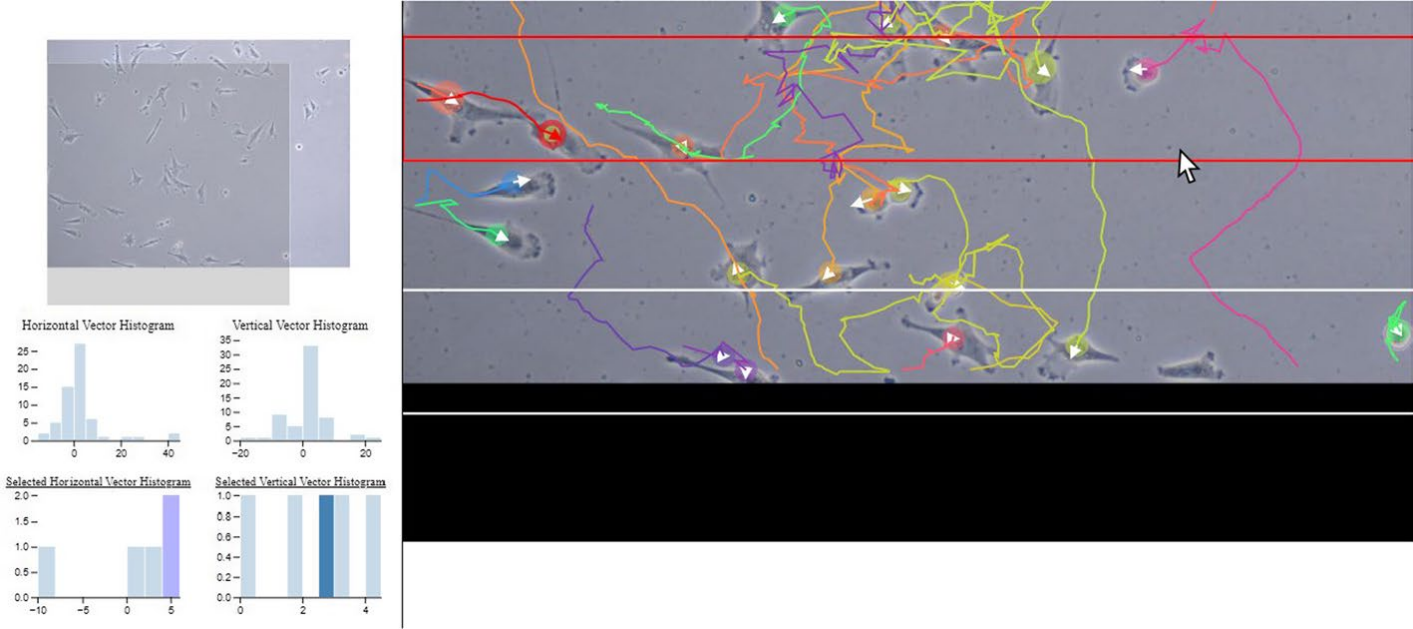
Analysis of cell migration (durotaxis)

In addition to the underlying visualization, directions of cell velocity vectors in a selected region of interest were reported using Split mode, which can also be useful for us to analyze the local stiffness within a linear stiffness gradient environment. Specifically, we were interested in analyzing the gradient stiffness via the durotaxis response of cells in the case. This effect is caused by cells tending to migrate to regions with higher stiffness gradients [20]. To perform this analysis, we divided the entire region in the trajectory view into vertical sub-sections (see Fig. 9). After selecting an area (highlighted red), we observed both horizontal and vertical vector histograms (of each time step) displayed instantly in the statistic view for the global (whole population) as well as the local (selected) region in the frame. Upper histograms in the statistic view showed Gaussian distributed cell migration direction when all cells were included. In contrast, cells in the chosen range showed a more vertical movement toward the positive values (according to lower histograms), indicating migration toward the stiffer area (a phenomenon known as durotaxis).

### Discussion

CellTrackVis has full potential with multiple viewpoints. In addition to the introduced usage scenario, one use case is the users can conveniently visualize different tracking results using some browsers, simultaneously, for a quick comparison of different tracking algorithms. However, the current version of software focuses on simultaneous visualization during the acquisition of time lapse and lacks further analysis and exportation of data and figures. Those are one of the main tasks that we are currently developing for the ‘Analysis suite’ version that can be compatible with this tool. Aforementioned shortcomings also will be addressed in consultation with experts in cell migration field.

Nevertheless, the proposed visualization system achieves the easy use by removing complicated steps which actively discourage beginners and sometimes experts as well. Although our work is not an all-around tool by one effort, its instant visualization provides a great tool for cell biologists to quickly interpret cell behaviors and ancestries even with some morphological insight, and for newcomers to easily start studying cell biology. Consequently, CellTrackVis allows various users to attain a more efficient decision-making process for the next experiments.

## Conclusions

CellTrackVis is an open-source visual analysis tool for studying moving live cells. Particularly, well-thought-out interactive views simply cover biological experiments by providing instant and straightforward visualization. Moreover, users can investigate cell movement patterns and lineages without wide-ranging knowledge thanks to the user-friendly design. Our self-reliant system is naturally browser-based and thus shovel-ready for online services as well. Source codes for CellTrackVis and its tutorial are freely available on GitHub pages with real data (MDA-MB-231), synthetic data [17], and relevant information. Future works include developing full 3D views, morphological analysis, and other visualization capabilities in adaptively size-changeable views. They will also include tools to correct tracking results through visual examination. Together with state-of-the-art cell tracking software, all of these features form an all-in-one ecosystem that supports efficient analysis in cell biology.

## Availability and requirements

Project name: CellTrackVis

Project home page: https://github.com/scbeom/celltrackvis

Operating system(s): macOS, Linux or Windows

Programming language: Python and JavaScript

Other requirements: Python 3.7+

License: GNU General Public License v3.0

Any restrictions to use by non-academics: no restrictions.

## Supplementary Information

**Additional file 1**. Visualizations using CellTrackVis on data sets of cell tracking challenge

## Data Availability

CellTrackVis and data sets are available at https://github.com/scbeom/celltrackvis. The tutorial is provided at https://scbeom.github.io/ctv_tutorial.

## Abbreviations

CSV: Comma-separated values
MOT: Multiple object tracking

## Footnotes

1 https://motchallenge.net/

2 http://celltrackingchallenge.net/
