# Supplementary material for: CellTrackVis: interactive browser-based visualization for analyzing cell trajectories and lineages
Source: BMC Bioinformatics. 2023 Mar 29;24:124. doi: 10.1186/s12859-023-05218-y (PMC10053428; doi:10.1186/s12859-023-05218-y)
Supplement: Supplementary file 1 — Additional file 1. Visualizations using CellTrackVis on data sets of cell tracking challenge [file 12859_2023_5218_MOESM1_ESM.pdf]

Supplementary materials for CellTrackVis: interactive browser-based visualization for analyzing cell trajectories and lineages

Changbeom Shim, Wooil Kim, Tran Thien Dat Nguyen, Du Yong Kim, Yu Suk Choi and Yon Dohn Chung

---

We provide additional figures by visualizing well-known data of cell tracking challenge[1] using CellTrackVis. There are 10 data sets (2D+time), and each set consists of training and challenge data. Since the proposed method is not a cell tracker, we use ground truths, i.e., training data, for the visualizations of cell trajectories, lineages, and images. Quantified information (statistics) is not included in the data sets. Snapshots are plotted in Figures 1 - 10.

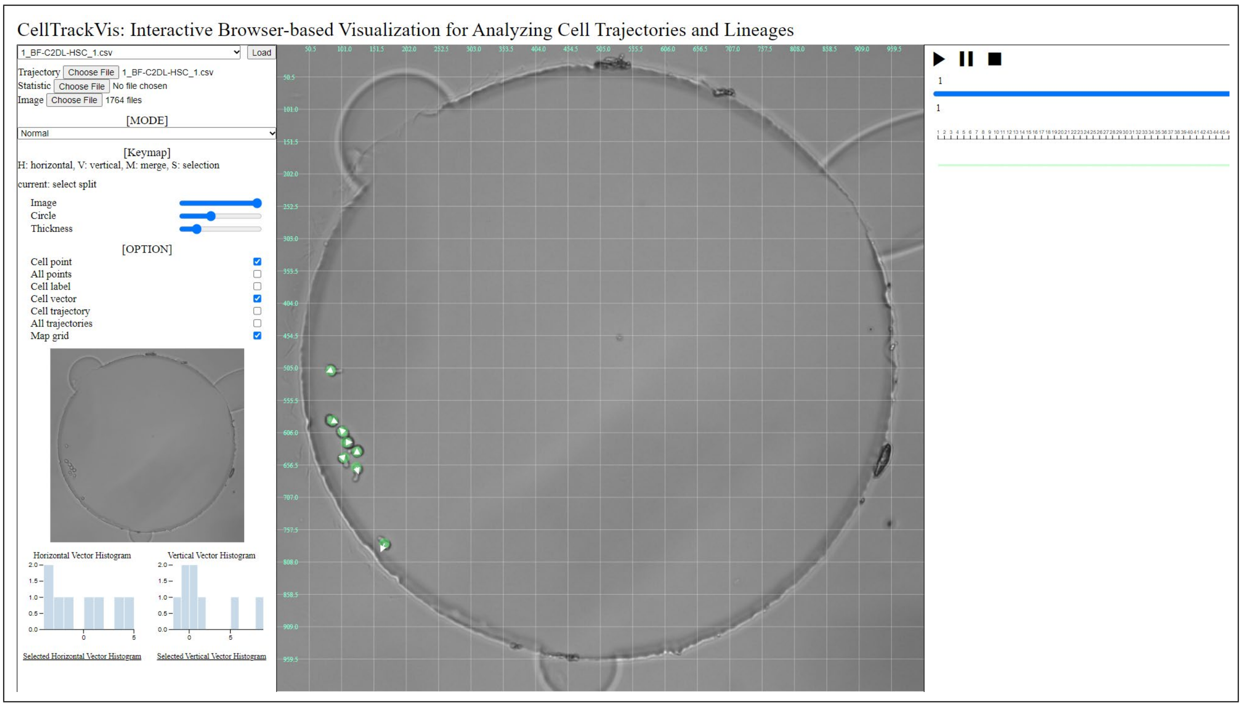

Figure 1: Visualization using CellTrackVis on mouse hematopoietic stem cells in hydrogel microwells (BF-C2DL-HSC).

[1] http://celltrackingchallenge.net/

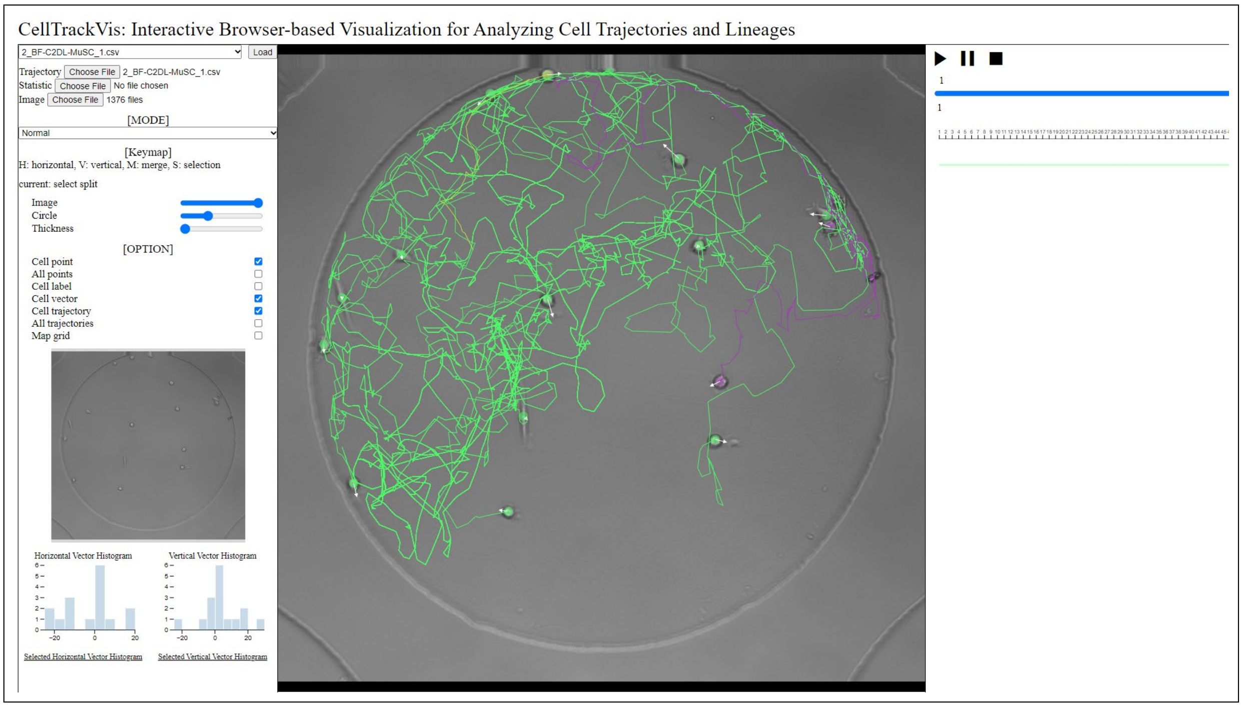

Figure 2: Visualization using CellTrackVis on mouse muscle stem cells in hydrogel microwells (BF-C2DL-MuSC).

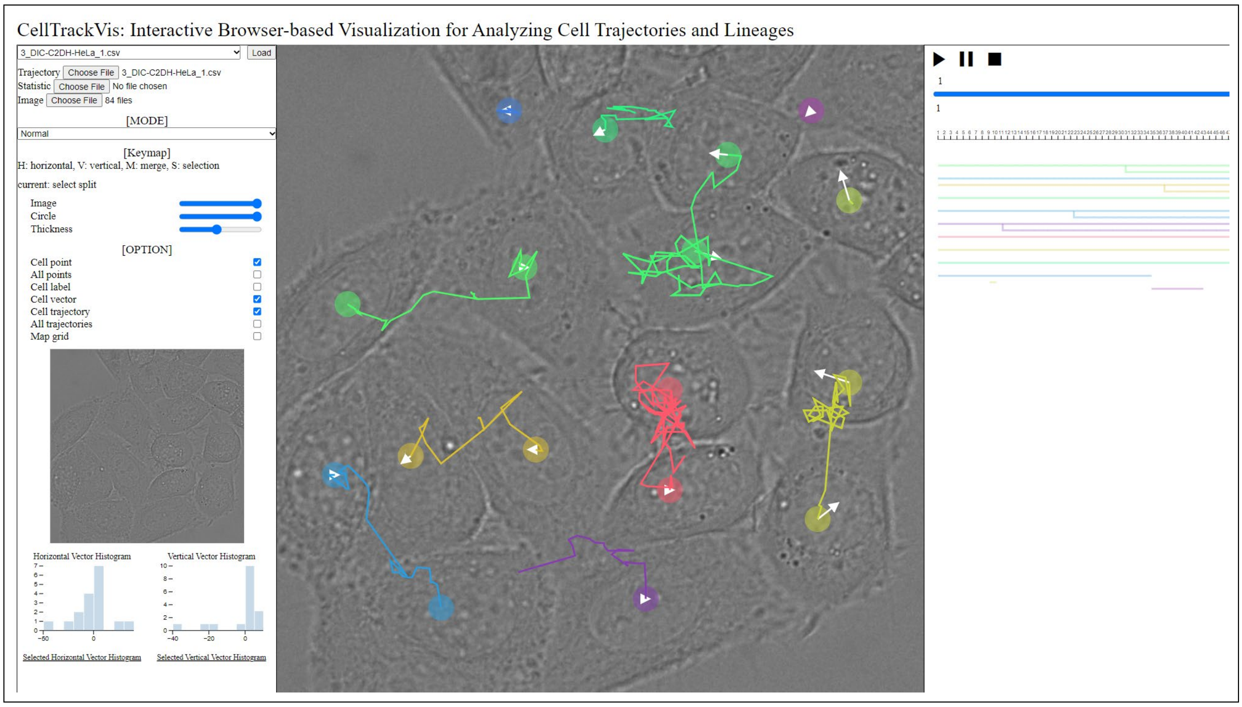

Figure 3: Visualization using CellTrackVis on HeLa cells on a flat glass (DIC-C2DH-HeLa).

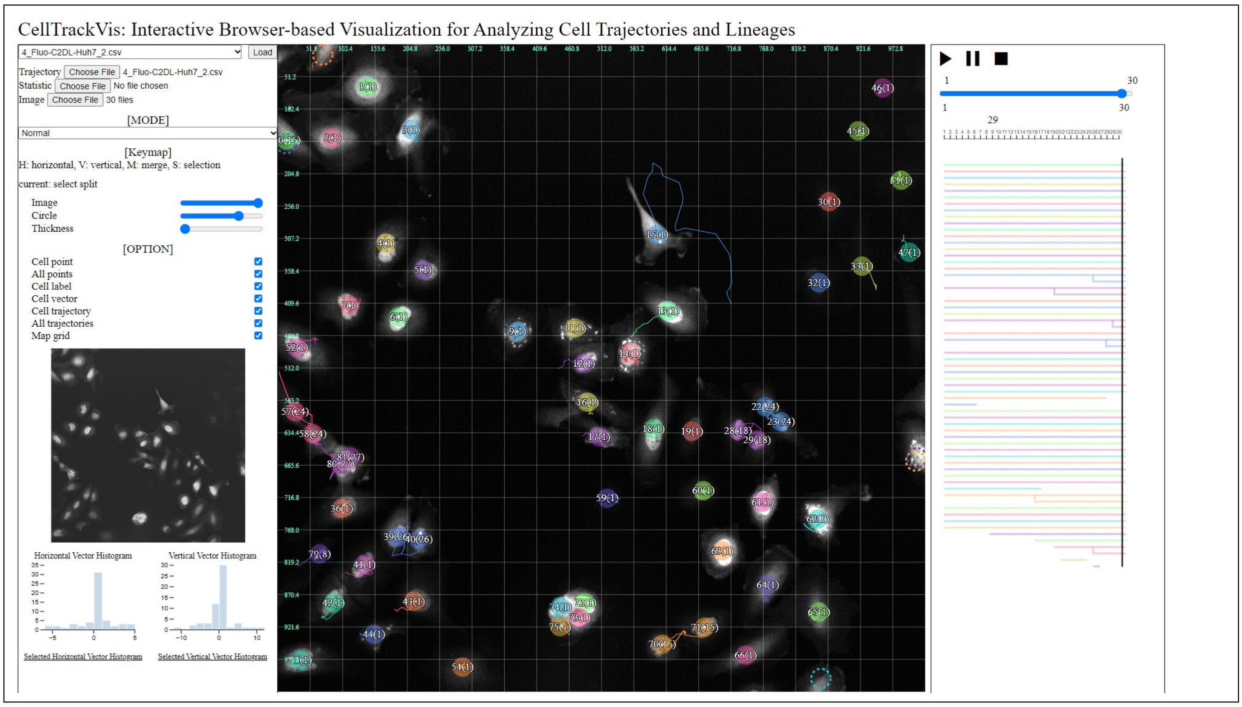

Figure 4: Visualization using CellTrackVis on human hepatocarcinoma-derived cells expressing the fusion protein YFP-TIA-1 (Fluo-C2DL-Huh7).

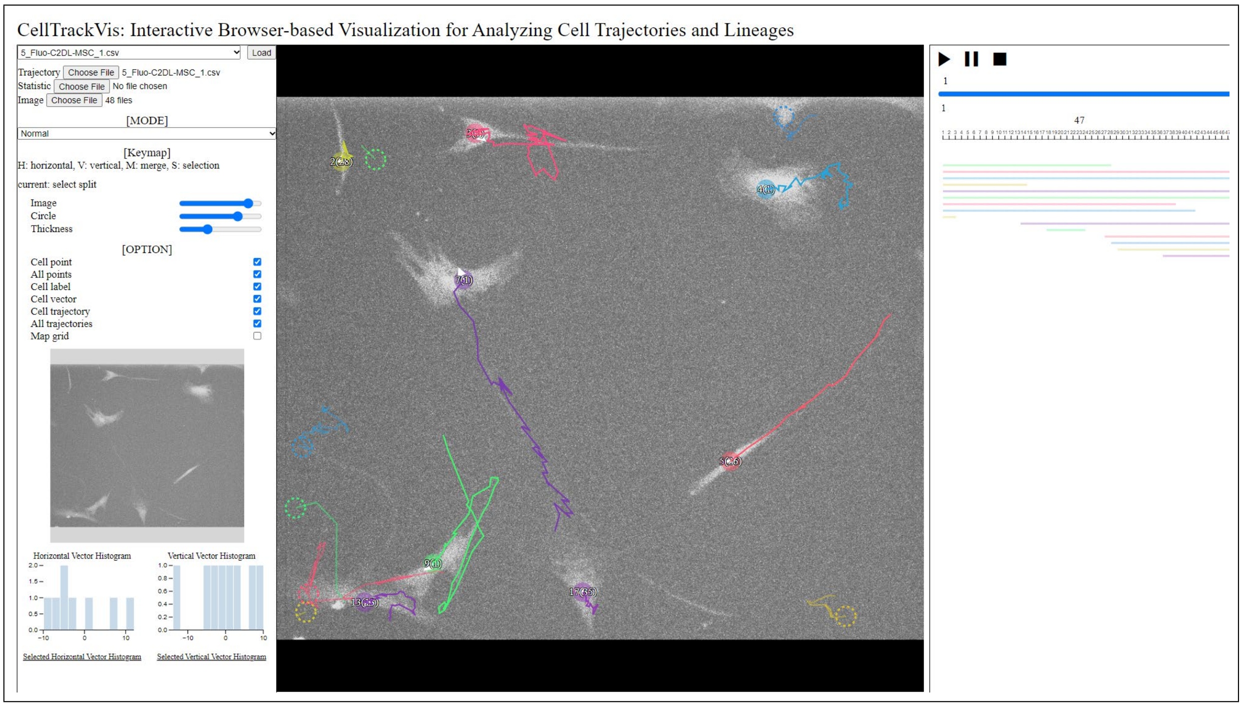

Figure 5: Visualization using CellTrackVis on rat mesenchymal stem cells on a flat polyacrylamide substrate (Fluo-C2DL-MSC).

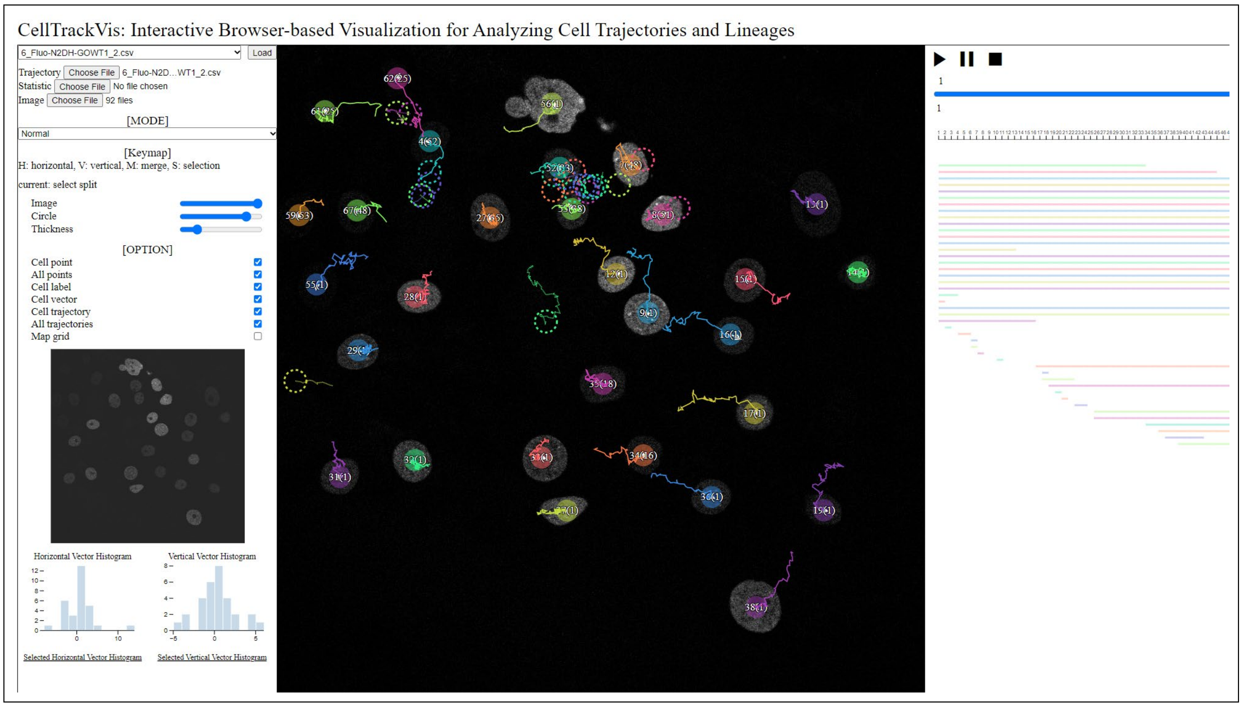

Figure 6: Visualization using CellTrackVis on GFP-GOWT1 mouse stem cells (Fluo-N2DH-GOWT1).

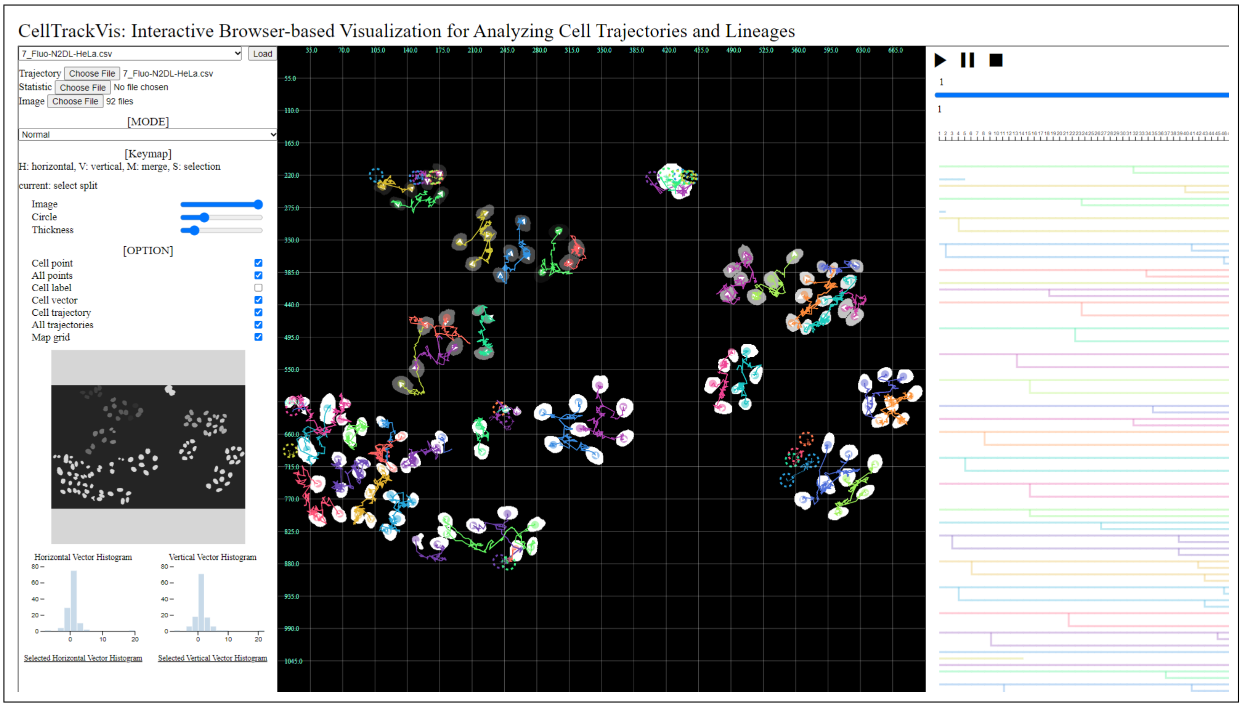

Figure 7: Visualization using CellTrackVis on HeLa cells stably expressing H2b-GFP (Fluo-N2DL-HeLa).

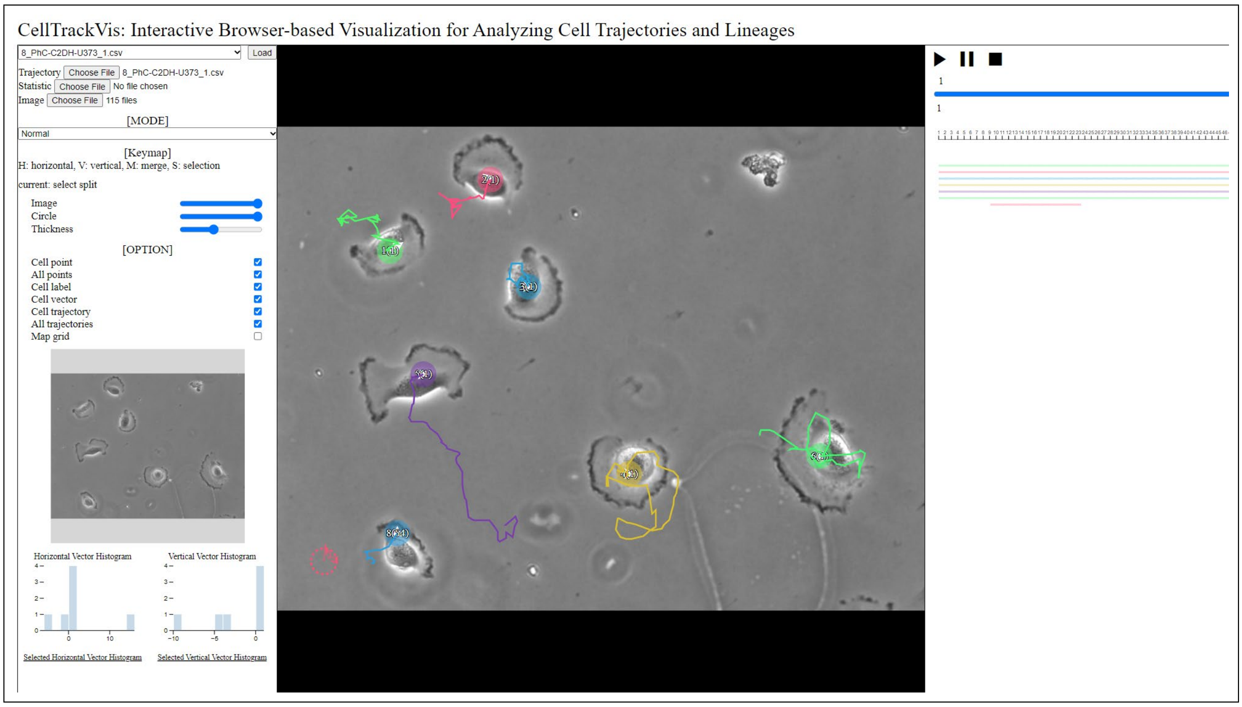

Figure 8: Visualization using CellTrackVis on glioblastoma-astrocytoma U373 cells on a polyacrylamide substrate (PhC-C2DH-U373).

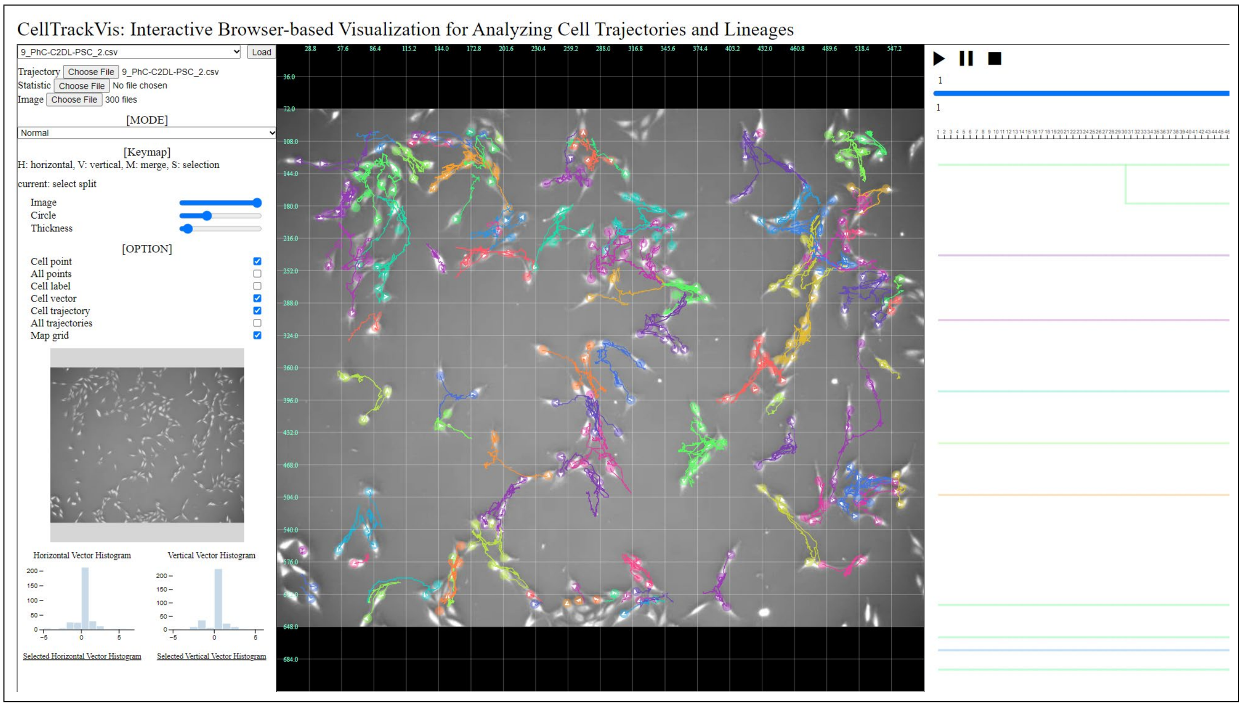

Figure 9: Visualization using CellTrackVis on pancreatic stem cells on a polystyrene substrate (PhC-C2DL-PSC).

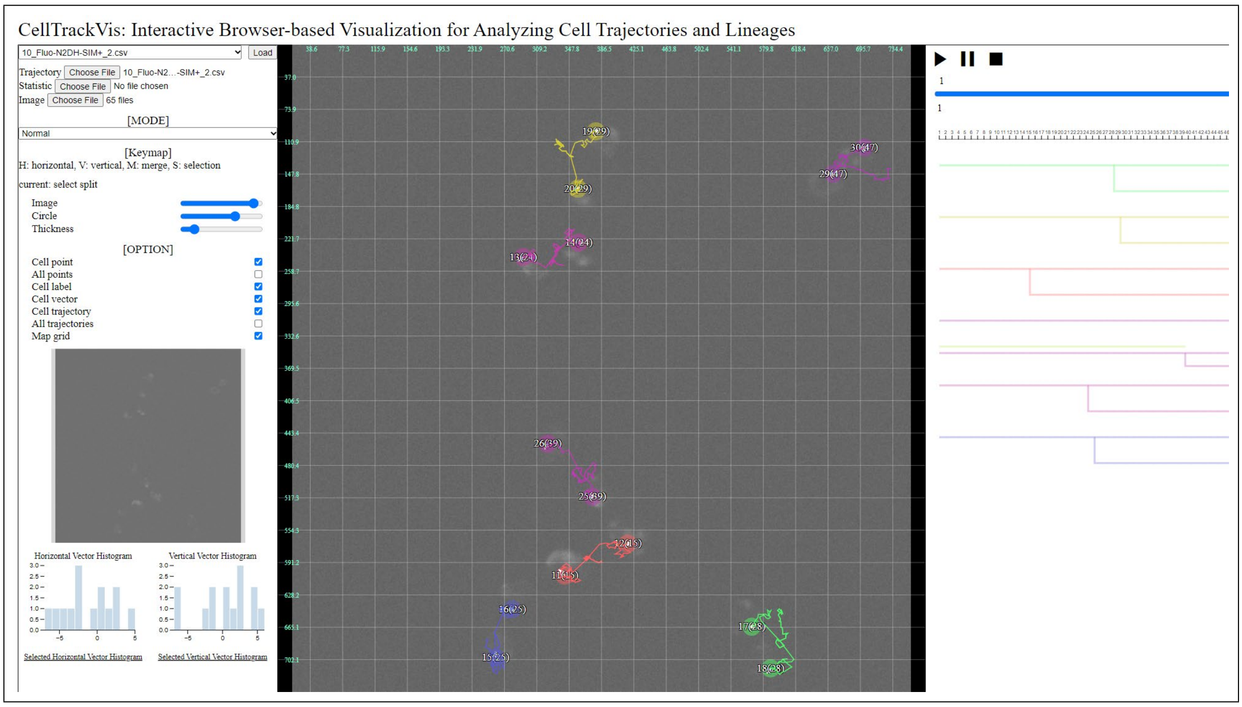

Figure 10: Visualization using CellTrackVis on simulated nuclei of HL60 cells stained with Hoescht (Fluo-N2DH-SIM+).
